# Privacy Concerns About Sharing General and Specific Health Information on Twitter: Quantitative Study

**DOI:** 10.2196/45573

**Published:** 2024-01-12

**Authors:** Pouyan Esmaeilzadeh

**Affiliations:** 1 Department of Information Systems and Business Analytics, College of Business Florida International University Miami, FL United States

**Keywords:** concern for information privacy, CFIP, peer privacy concern, PrPC, health information disclosure, Twitter, empirical study

## Abstract

**Background:**

Twitter is a common platform for people to share opinions, discuss health-related topics, and engage in conversations with a wide audience. Twitter users frequently share health information related to chronic diseases, mental health, and general wellness topics. However, sharing health information on Twitter raises privacy concerns as it involves sharing personal and sensitive data on a web-based platform.

**Objective:**

This study aims to adopt an interactive approach and develop a model consisting of privacy concerns related to web-based vendors and web-based peers. The research model integrates the 4 dimensions of concern for information privacy that express concerns related to the practices of companies and the 4 dimensions of peer privacy concern that reflect concerns related to web-based interactions with peers. This study examined how this interaction may affect individuals’ information-sharing behavior on Twitter.

**Methods:**

Data were collected from 329 Twitter users in the United States using a web-based survey.

**Results:**

Results suggest that privacy concerns related to company practices might not significantly influence the sharing of general health information, such as details about hospitals and medications. However, privacy concerns related to companies and third parties can negatively shape the disclosure of specific health information, such as personal medical issues (β=−.43; *P*<.001). Findings show that peer-related privacy concerns significantly predict sharing patterns associated with general (β=−.38; *P*<.001) and specific health information (β=−.72; *P*<.001). In addition, results suggest that people may disclose more general health information than specific health information owing to peer-related privacy concerns (*t*_165_=4.72; *P*<.001). The model explains 41% of the variance in general health information disclosure and 67% in specific health information sharing on Twitter.

**Conclusions:**

The results can contribute to privacy research and propose some practical implications. The findings provide insights for developers, policy makers, and health communication professionals about mitigating privacy concerns in web-based health information sharing. It particularly underlines the importance of addressing peer-related privacy concerns. The study underscores the need to build a secure and trustworthy web-based environment, emphasizing the significance of peer interactions and highlighting the need for improved regulations, clear data handling policies, and users’ control over their own data.

## Introduction

### Background

Reports and analyses highlight that approximately 60% of health-related tweets contain links to health-related websites [1]. Twitter users frequently share health information related to chronic diseases, mental health, and general wellness topics [2]. Twitter is a popular platform for health-related conversations because it allows users to share their thoughts, experiences, and information in real time [3]. This platform can be particularly useful for sharing information about health events, such as disease outbreaks or public health campaigns. In addition, Twitter can be used to connect with others with similar health concerns or interests and to access information from health care professionals and organizations [4]. Twitter is a social media platform with a character limit, which makes sharing detailed information about health issues difficult. Moreover, the information shared on Twitter may not always be accurate or reliable, as it is not always fact-checked or verified. However, Twitter is a common platform for people to share opinions, discuss health-related topics, and converse with a wide audience. According to a survey conducted by the Pew Research Center, 21% of Twitter users have used the platform to share information about a health condition [5]. The survey also found that 20% of Twitter users have followed a health organization or medical professional on the platform, and 15% have searched for information about a health condition on Twitter. Users often express their views about health policies, medical breakthroughs, health care services, and public health issues [6]. Health professionals, researchers, advocacy groups, and patients actively participate in these discussions, contributing diverse perspectives and sharing evidence-based information. This open and rapid exchange of ideas allows health information dissemination and facilitates conversations that can influence public opinion and policy decisions [7].

Sharing health information on Twitter raises privacy concerns as it involves sharing personal and sensitive data on a public platform [8]. Information privacy refers to individuals’ control over collecting, using, and disclosing their personal information. Regarding sharing personal health information, web-based information privacy refers to protecting sensitive health data from unauthorized access, secondary use, or disclosure [9]. It involves ensuring that individuals can make informed decisions about how their health information is shared and used and that appropriate safeguards are in place to protect the confidentiality and security of this information. The potential privacy risks associated with sharing health information on Twitter can be grouped into 3 reasons. First, potential identification and disclosure of personal information—sharing health information on Twitter can inadvertently lead to disclosing personally identifiable information. A study found that anonymized data from health-related tweets could be reidentified to reveal the identity of users [10]. Researchers were able to reconstruct personal health stories and connect them to specific individuals, highlighting the potential privacy risks involved. Second, data mining and analytics—third parties can analyze and use health-related tweets for various purposes, including targeted advertising or creating consumer profiles. Researchers analyzed tweets related to mental health and found that the content could be used to predict users’ self-reported diagnoses, medication use, and other personal information [11]. This demonstrates the potential for extracting sensitive health-related data from Twitter. In addition, a study used deep neural networks to identify personal health experience tweets, highlighting the potential for using Twitter as a data source for health surveillance studies [12]. Third, public disclosure of sensitive health information—sharing health information on Twitter might inadvertently expose individuals to public scrutiny and judgment. A study examined tweets related to mental health and found that users often disclosed personal experiences, symptoms, and treatments [13]. Although this sharing can provide support, it can also expose individuals to potential stigma, discrimination, or unwanted attention.

Previous studies suggest that individuals may be comfortable with sharing general information that is not sensitive on social media [14]. However, people may not be likely to share personal information, especially health-related data, owing to privacy concerns [15]. According to previous studies, privacy concerns can arise from companies’ information collection and use policies in the age of medical big data [16] and web-based social interactions that may threaten information privacy [17]. Twitter is reported as an important data set for vendors, researchers, and medical companies to collect health-related information [18]. Many medical companies collect health information and patient experiences from Twitter for big data analysis to find patterns for public health management [19]. Although big data collection and data mining techniques could help generate intelligence for monitoring public health issues, they can cause privacy concerns. Reports highlight that many Twitter users have experienced invasion of privacy owing to companies’ collection, sharing, and analytics practices that use information from their tweets, including private health information [20].

Although there are various studies of vendor-related privacy concerns [21] and peer-related privacy concerns [22], little is known about whether these 2 aspects of privacy concerns may collectively influence information-sharing behaviors. As privacy violations can be related to peers (such as inappropriate comments and unauthorized retweeting) and companies (sharing personal information with third parties), more studies are required to examine whether information-sharing disclosure can be affected equally by vendor-related and peer-related privacy concerns. In this study, we aimed to determine whether both aspects of privacy concerns (ie, concern for information privacy [CFIP] and peer privacy concern [PrPC]) can mutually change health-related information-sharing decisions or whether one’s effects can dominate or overshadow the impact of the other. For instance, whether the nature of the relationships and contexts with 2 different information trustees (ie, vendors and peers) can influence information dissemination behavior. Thus, we argue that both aspects of privacy concerns should be considered in a model to better characterize information privacy on social media. Investigating the importance of privacy concerns related to companies and vendors (such as Twitter analytics) and web-based peers (such as retweeting a focal user’s health information without permission) in case of disclosing public and private health information on Twitter would be the main contribution that makes this study different from others.

This argument is built based on 4 reasons. First, although the main interactions on social media are mainly peer oriented, vendors can still collect a lot of personal data (such as health information) without authorization and use it for unconsented purposes [23]. There have been instances of social media platforms, including Twitter, being used by organizations for health-related data mining and analysis [24]. Twitter data can provide valuable insights into health-related trends for health care organizations through analytics [25]. Researchers and companies (such as pharmaceutical manufacturers) have used Twitter data to track and analyze health-related trends, including disease outbreaks, medication use, and public health concerns [26]. For example, a study found that Twitter data could be used to track the spread of influenza and predict outbreaks [27]. Another study uncovered that Twitter data could be used to monitor adverse drug reactions and identify potential safety concerns [28]. Pharmaceutical companies have also used Twitter data to monitor medication use and patient experiences. For example, a study reported that Twitter data could be used to monitor patient experiences with antidepressant medications [29]. Moreover, it is common for organizations and vendors, including those in the health care industry, to monitor social media platforms to gather insights about consumer opinions, preferences, and trends [30]. Twitter, as a popular social media platform, has been used for these purposes [31]. Health care organizations and vendors may collect health-related information, such as discussions about medical conditions, treatment experiences, and patient preferences, from public Twitter profiles [19]. These insights can be valuable for marketing and market research purposes.

Second, although the primary purpose of peer-to-peer (P2P) interactions on social media is to maintain social connections with peers and there are no explicit business-to-consumer interactions, companies can still use social media analytics to investigate published health information. Companies can leverage various analytics tools to gather and find meaning and patterns in data collected from social channels to support business decisions (such as predicting the risk factors to manage public health) [32]. Third, individuals share a variety of information (such as about lifestyle, health status, chronic issues, and medication) on social media, which can be more sensitive than conventional e-commerce information (such as transaction records). Disclosing a wide range of information across social media platforms could raise concerns about whether companies and peers misuse the shared data (eg, health information). Fourth, Web 2.0, a fundamental technology supporting social media, mainly focuses on bilateral relationships between peers. However, it does not remove traffic between companies and social media users. Thus, peers can comment on conversations about a user’s health condition and share others’ personal health information on their own channels. In contrast, companies can use big data analytical tools to collect, use, or share users’ personal health data with third parties.

### Study Objectives

The main objective of this study was to investigate the concept of information privacy concerns in the context of social media based on both vendor-related and peer-related aspects. To do so, we used the survey research methodology and Twitter as the empirical context. We also relied on theories discussing 2 dimensions of information privacy (ie, CFIP and PrPC) as the theoretical foundation of our proposed model. In this study, CFIP, emphasizing both consumer perspectives and company responsibilities, represented privacy concerns related to web-based practices of companies and vendors (such as collection and sharing of self-shared information), and PrPC referred to privacy concerns about losing control over digital communications and web-based interactions with peers. Thus, we suggest that information privacy on social media can be multidimensional, focusing on privacy violations associated with companies’ (vendors’) practices and sharing behaviors of peers. The integration of CFIP and PrPC can comprehensively present the entirety of privacy concerns about web-based health information. This study contributes to both theory and practice. We shed more light on information privacy conceptualization in the context of social media. This study also provides an interactive outlook and practical recommendations for handling privacy issues by explaining how web-based vendors and peers may cause privacy violations when dealing with health information (general and specific) shared over the web.

### Variable Conceptualization, Theoretical Foundation, and Research Hypotheses

#### General and Specific Health Information

Individuals can use web-based channels to share general health information, such as information about treatments, medications, side effects, hospitals, medical costs, and healthy behaviors [33]. For instance, people are likely to tweet about general obesity-related topics, such as the relationship between fast food and weight gain [34]. Another study identifies general tobacco-related tweets (such as information about smoking, cigarette risks, and quitting) as the primary conversational data sets for health-related topics on Twitter [35]. Moreover, people can use tools such as Twitter to share specific health-related information, including past medical history, allergies, personal medications, private health issues, and signs and symptoms. For example, a study indicates that people disseminate information about diagnoses, advice based on personal experience, use of specific medications, side effects, negative reactions, and treatments on Twitter [31]. Another study highlights that people use Twitter to share their COVID-19–related symptoms and personal health issues during the early stages of the pandemic [36].

Sharing public and private health information can be valuable for web-based peers and affect their health-related decisions. General information can enable web-based users to find some facts about hospitals, physicians, and diseases. Disseminating specific information can share important insights and advice based on personal health conditions, medical treatments, care planning, and medical experiences with chronic diseases. General health information can be publicly available regardless of personal experiences. However, specific health information can be unpleasant to share because it may contain more private information. As health information dissemination has 2 sides, questions still remain as to what dimensions of information privacy may strongly affect sharing behaviors on Twitter.

#### CFIP Constructs

There is evidence suggesting that companies use tweets to collect health information. For example, reports show that public health researchers use Twitter data to study the world’s health. A recent study indicates that the amount of textual health-related data, which could be personal, collected by various organizations is growing (especially during the COVID-19 pandemic) [37]. Another study argues that health care researchers and research companies have used social media data sources such as Twitter to study public health [19]. Owing to the importance of the Twitter database, the Centers for Disease Control and Prevention (CDC) designed a document to guide employees and contractors on using Twitter to disseminate health information and engage with individuals and partners [38]. A study indicates that companies increasingly use Twitter to share public health information and collect real-time health data using crowdsourcing methods [39]. Information privacy, which refers to people’s ability to control their information, is essential in e-commerce and social media [40]. Several studies explain the privacy concerns specific to the mobility data collection context [41]. Thanks to emerging technology (such as Web 2.0), protecting personal information has become a growing concern for web-based users. CFIP is a general concern about how organizations can use and protect consumers’ information [21]. CFIP explains concerns about organizations’ information collection practices, use policies, and access to consumers’ personal information [42]. Previous studies indicate that examining consumers’ concerns about how companies (vendors) may use their personal information significantly affects their willingness to engage in web-based transactions actively [43].

In this study, following most previous studies, CFIP is posited as a multidimensional construct with 4 dimensions to measure individuals’ concerns about organizations’ information privacy practices [44]. Collection pertains to individuals’ concerns about what web-based information is collected and whether such information is stored properly. Unauthorized secondary use explains individuals’ concerns about whether the information collected for a consented purpose may be unethically and illegally used for other purposes without obtaining authorization. Improper access implies individuals’ concerns about whether unauthorized people (entities) can access, view, and share their information. Finally, concerns about errors reflect whether individuals’ information is appropriately protected to minimize accidental or intentional errors [44]. Therefore, the multidimensional scale of CFIP reflects the complexity of individuals’ privacy concerns [21]. According to Stewart and Segars [40], CFIP is developed as a second-order construct with 4 reflective first-order factors. In this study, we also considered CFIP as a high-order construct with reflective factors. The logic behind conceptualizing this construct as reflective was that the privacy concerns related to companies are reflective of the 4 dimensions (ie, collection, unauthorized access, errors, and secondary use) and the expected interactions among them. Therefore, these dimensions can reflect the same theme and may covary.

Although sharing information on Twitter is more oriented toward interactions with web-based peers, privacy concerns about the collection and misuse of digitized health information by vendors and companies still remain significant. Previous studies provide strong evidence suggesting that web-based users of Twitter are concerned about several aspects of their information privacy, from collection of a lot of data to misuse [45]. Our study focused on individuals’ perceptions about general CFIP owing to policies and practices of vendors and organizations that may collect, access, and use health information shared on Twitter rather than concerns about a particular vendor. According to the four dimensions of the CFIP construct, individuals who demonstrate high privacy concerns believe that (1) a lot of health information is collected by organizations from users’ Twitter accounts, (2) such health information is not appropriately protected against possible errors, (3) various organizations may use health-related information on Twitter for other purposes without authorization (such as data mining, surveillance, research, and business intelligence), and (4) there is lack of visibility into accurate security measures to control who can access and use health information from tweets.

Thus, the CFIP construct can be extended to privacy concerns about a wide range of vendors and companies accessing and using tweets containing health information. This concern is not the same as privacy issues owing to interactions with a specific vendor in the context of e-commerce (such as retail platforms). In these conventional interactions, privacy concerns may focus on personal, factual information shared in web-based transactions and services (such as demographic information). However, CFIP in the social media domain deals with concerns associated with the following uncertainty: which organizations collect personal posts, which unauthorized entities can view and share information, why and how the information is used (for instance, data mining), and how the information is protected from internal and external errors and misuse. Therefore, we argue that CFIP cannot be ignored in examining information privacy in social media because users may not have direct relationships with organizations on these digital platforms, but they are still concerned about how their posts can be collected and misused by various companies.

Sharing general health information could indicate a user’s rich medical information and wealth of medical knowledge. In contrast, sharing specific health information can show that the user may want to contribute or seek informational and emotional support by disseminating personal experiences and medical history. However, when privacy concerns about the collection and misuse of shared data by organizations are not addressed, users are not likely to disseminate general or specific health information on Twitter. Moreover, we can expect that because specific health information is more sensitive and private, web-based users may generally become more cautious about sharing it. Therefore, we hypothesized the following:

- Hypothesis 1A (H1A): CFIP negatively influences general health information dissemination on Twitter.
- Hypothesis 1B (H1B): CFIP negatively influences specific health information dissemination on Twitter.
- Hypothesis 1C (H1C): CFIP has a more negative effect on specific health information dissemination than on general health information sharing on Twitter.

#### PrPC Constructs

Owing to the nature of Web 2.0, users can communicate, create content, and share it via communities, social networks, and virtual worlds [46]. Web-based users can share a wide range of information and experiences on social media. The information can be objective (based on factual data) or subjective (based on personal interpretation, feelings, tastes, or opinions) [47]. The range can start with demographic information (eg, age, gender, and race); continue with political views, humanitarian opinions, and health information; and end with comments on others’ posts [48]. People can use different formats, such as text, pictures, and videos, to disseminate information. Peers are important components of social networks; however, they can threaten information privacy through inappropriate sharing behaviors and unintended consequences of web-based interactions [49].

Web-based transactions with peers on social media affect users’ decisions about whether they want to reveal their personal information (such as feelings and likes) and create an image consistent with their personal identity [50]. In this study, peers could be web-based friends who may have long-lasting and affect-laden connections with a user and any web-based users who interact through social media channels. Previous studies highlight the importance of PrPCs in the context of web-based interpersonal relationships where other peers can access and view a user’s web-based information [51]. Peer-related privacy refers to possible risks of privacy invasion because of direct and indirect web-based interactions with peers [17]. Social bots and fake and spam accounts can also raise privacy violation risks by potentially exposing several peers to a focal user’s posts using machine learning algorithms [52]. Previous studies indicate the threat of using social bots on social networks, increasing the likelihood of privacy breaches where even more private user data are exposed [53]. Understanding who can access web-based information (such as a post related to signs and symptoms of depression) and with whom such information is shared can significantly raise privacy concerns. For example, a study shows that sharing information with only selected friends in social networking services perceived higher control than sharing information with all friends [54].

Thus, information-sharing behaviors on social media may erode the ability of users to control their virtual space and personal boundaries. Leaving an inappropriate comment for a user who posted about *seeking ways to lose weight*, can increase privacy concerns about lack of control to maintain the privacy of their Twitter space. A study posits that managing the privacy of virtual territory refers to defining the level of access to and interaction others can have within a user’s territory (eg, allowing peers to see or comment on the post) [55]. Peers can also play a bilateral role in web-based social interactions. They can intentionally or unintentionally share a user’s personal health information with others and expose the user to others’ personal information that they might not like to view. The user may think that if others’ personal health information has been shared with me, my posts can also be revealed to others. Thus, communication privacy can significantly affect how individuals and relational parties share private information on social media [56].

A recent study defines PrPC as the sense of inability to control personal boundaries in web-based interactions owing to web-based peers’ behaviors [22]. They describe this term using 4 reflective dimensions: peer-related information privacy, psychological privacy, virtual territory privacy, and communication privacy. Peer-related information privacy denotes concerns about who can see what type of information and when and how such information is disclosed to other web-based peers. For posts shared by a user, the main concern is unauthorized access and secondary use of data by other peers. On Twitter, this can happen through retweeting and commenting. Peers can also initiate posts or conversation threads to disclose a user’s personal information without authorization. A privacy concern is about the accuracy of personal information shared by peers. Thus, peers’ sharing can be a source of private information leaks.

Psychological privacy explains the control over input information coming from others to shape feelings, opinions, and beliefs. Information sharing is 2-way traffic in social media (ie, from a user to peers and from peers to a user) [57]. As people are exposed to posts shared by celebrities, business magnates, politicians, and other web-based users, their behaviors and opinions are increasingly affected by input information from peers. Peers on social media can influence users’ behavior by applying social influence through public comments on posts [58]. Privacy concerns become more intense when users’ opinions and psychological independence are intentionally manipulated by social bots [59]. In this situation, users are not able to make a decision independent of other web-based peers’ ideas. Moreover, receiving a lot of unwanted information from peers may influence value systems, attitudes, identities, and choices.

Virtual territory privacy represents concerns about an individual’s inability to achieve control over other peers’ interactions with their virtual properties (such as Twitter accounts) and shared conversations (postings). Previous studies suggest that the sense of ownership and emotional attachment to personal territory can be generalized to the social media domain [60]. Similar to other personal belongings, virtual properties are seen as private. Thus, any unwanted addition to or revision of personal information can be considered as an intrusion, which may increase privacy violation risks [45]. Finally, communication privacy reflects an individual’s lack of control over how and when other peers can make direct web-based conversations. For example, peers may use various communication tools to engage individuals in a group conversation about potentially embarrassing or stigmatic health-related topics. Then, users may feel pressured by being involved in such undesirable conversations with unfamiliar people.

Individuals may become more likely to share general or specific health information on Twitter when they think it is useful for other web-based peers (eg, they can make better medical decisions). However, peer-related concerns may prevent them from disseminating such information. Peers are participants in social media and can freely collect and share information that is sometimes considered as unwanted interference. For instance, if peers retweet a post containing personal information about postsurgery recovery plans without authorization or tag a user who posted general educational content about HIV, these web-based interactions may violate privacy needs and raise privacy concerns. In return, users may change the pattern of health information dissemination and become more cautious in sharing medical facts or personal experiences. Thus, we formulated the following hypotheses:

- Hypothesis 2A (H2A): PrPC negatively influences general health information dissemination on Twitter.
- Hypothesis 2B (H2B): PrPC negatively influences specific health information dissemination on Twitter.
- Hypothesis 2C (H2C): PrPC has a more negative effect on specific health information dissemination than on general health information sharing on Twitter.

### CFIP and PrPC Are Privacy Concerns for Twitter Users

Although tweets are publicly accessible by default, users likely expect some degree of privacy and control over their personal health information shared on the platform. Previous literature has found that even when posting content publicly on social media, individuals still have privacy interests and concerns about how their data might be used or accessed [61]. General health information shared publicly on Twitter, such as mentions of hospitals, physicians, and common diseases, is not considered protected or private. However, more specific personal health details, such as past medical history, allergies, medications, and current symptoms, could reveal private information about an individual’s health status. Although these details may be shared publicly by default on Twitter, users likely still have privacy concerns about this content being widely disseminated or used without their consent.

The concepts of CFIP and PrPC capture these types of privacy concerns. Although users are voluntarily sharing health information publicly on Twitter, they may still desire control over how these data are accessed and used. CFIP reflects concerns about using or sharing personal health data by third parties such as researchers or companies without the user’s knowledge or permission. Even if users willingly post health information publicly, they may still desire control over how that data are collected, analyzed, or shared by entities such as researchers, pharmacies, insurance companies, and so on. PrPC represents concerns about controlling boundaries around health disclosures and limiting exposure to certain audiences, such as employers or insurers, who could misuse the information. Users must balance sharing personal details with managing social risks if the information reaches unintended viewers such as employers, family members, or friends. Thus, although Twitter data are technically public, users are likely to have nuanced privacy interests surrounding their health disclosures. Therefore, concepts such as CFIP and PrPC are useful for quantifying expectations regarding control, anonymity, and audience boundaries that persist even when posting health care–related content openly over the web.

### Difference Between the Conceptualization of CFIP and PrPC

#### Overview

We used an interactive approach to provide a holistic view of information privacy in the context of sharing health information on Twitter. Using this approach, this study actively engaged with the 2 aspects of privacy concerns (CFIP and PrPC) in a dynamic way, considering the interplay between them, as opposed to treating them as isolated, independent factors. Therefore, we examined how these 2 aspects of privacy concerns interact with each other and how this interaction affects individuals’ behavior on Twitter. It should be mentioned that the dimensions used for CFIP and PrPC may differ because of the different nature of the relationships and contexts involved. Although the underlying concept of privacy concerns remains the same, the specific dimensions or factors that contribute to CFIP and PrPC may vary owing to the distinct characteristics of vendors and peers as information trustees.

#### Role and Control

Vendors typically have a professional or business relationship with individuals, where they are entrusted with handling personal information for specific purposes (eg, health care providers and web-based retailers). In this context, individuals may be concerned about vendors’ control over their information; how it is collected, used, and shared; and the potential for data breaches or unauthorized access.

#### Trust and Reputation

CFIP dimensions often include factors related to trust and reputation, such as trustworthiness, perceived reliability, and credibility of vendors. As individuals rely on vendors to handle their personal information responsibly, dimensions related to trust and reputation become important for CFIP measurement.

#### Legal and Ethical Considerations

CFIP dimensions may also include factors related to legal and ethical considerations, such as compliance with privacy laws, informed consent, and transparency in data practices. Individuals may be concerned about whether vendors meet the legal requirements and ethical standards in protecting their health information.

In contrast, peers, who are individuals within an individual’s social network or community, may have different dimensions of privacy concerns. Social interactions, trust, reciprocity, and the potential for social consequences typically characterize peer relationship dynamics. Some factors that could influence PrPC dimensions include the following.

#### Social Norms and Expectations

PrPC dimensions may reflect concerns about social norms and expectations related to privacy within the peer group. Individuals may worry about how their health information might be perceived, shared, or used by their peers and the potential impact on their social relationships or reputation.

#### Social Influence and Peer Pressure

PrPC dimensions may capture the influence of peer pressure or the fear of negative social consequences. Individuals may be concerned about potential judgment, stigma, or discrimination based on their health information within their peer group.

#### Personal Boundaries and Intimacy

PrPC dimensions may include factors related to personal boundaries and the level of intimacy within peer relationships. Individuals may be concerned about the extent to which personal health information should be shared with peers and the potential impact on their privacy, autonomy, and self-disclosure.

Although the underlying concept of privacy concerns is present in both CFIP and PrPC, the dimensions may differ owing to the distinct characteristics and dynamics of the relationships involved. Thus, considering these differences when developing measurement instruments is important to accurately capture individuals’ concerns regarding privacy in different trust relationships.

### Research Model

The model focuses on health information and Twitter (as the research context). There are a few critical differences in the privacy concerns around health information compared with other types of information. First, health information is considered to be very sensitive and private. It can reveal details about medical conditions, treatments, prescriptions, family history, and so on. Other types of information, such as social media posts or shopping habits, are generally not as sensitive. Second, health information has strict legal protections such as Health Insurance Portability and Accountability Act in the United States and General Data Protection Regulation in the European Union. These laws place restrictions on how health data can be collected, shared, and used. Other information does not have the same level of legal safeguards. Third, health information could potentially be used to discriminate against people in areas such as employment, insurance, and so on. This type of discrimination is legally prohibited, but the risk remains owing to the sensitive nature of the data. Other data, such as social media posts, have less potential for this type of discrimination. Finally, breach of health information is considered very serious, given the sensitivity of the data. Strong security protections are needed, and breaches can carry heavy penalties. Breaches of other types of data may not have the same level of severity.

Regarding privacy on social media, there are some key characteristics of the concerns around Twitter compared with other platforms. First, most Twitter content is public by default, whereas other platforms such as Facebook allow more privacy controls. This can raise concerns about a lack of control over dissemination. Second, tweets are often archived and searchable indefinitely; therefore, there are concerns about permanent availability even for “deleted” content. Other platforms may have more ephemeral sharing. Third, the open nature of Twitter makes it easy for tweets to spread rapidly and become viral compared with platforms such as Instagram, where sharing can be more controlled. This raises concerns about loss of context and lack of containment. Finally, the ability to create anonymous accounts on Twitter is greater than that on platforms such as Facebook that require real identities. This raises concerns about harmful speech, misinformation, and so on.

**Figure 1:**
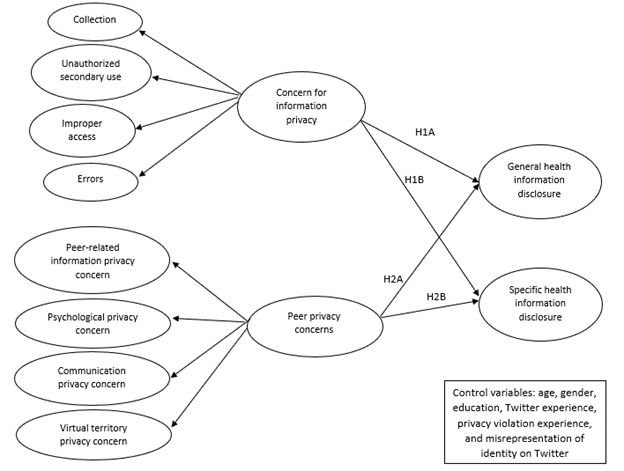
Research model. 1A: hypothesis 1A; 1B: hypothesis 1B; 2A: hypothesis 2A; 2B: hypothesis 2B.

We proposed the following research framework for disclosing general and specific health information on Twitter by integrating 2 aspects of information privacy concerns (Figure 1). As several studies may have found empirical evidence for the hypotheses proposed in this study, we need to clarify what is new in our study. First, this study integrated both aspects of privacy concerns for the first time in a model. Previous studies examined either privacy concerns related to companies’ practices with web-based information (CFIP) [62] or concerns related to the web-based behaviors of peers (PrPC) [63]. However, as mentioned in the previous section, individuals may be concerned about disseminating their health information on Twitter because companies’ collection practices and web-based peers’ behaviors could violate their privacy. In this study, we wanted to examine whether both aspects of privacy concerns (ie, CFIP and PrPC) can collectively change health-related information-sharing decisions or whether one can dominate the other. For instance, whether the nature of the relationships and contexts with 2 different information trustees (ie, vendors and peers) can shape information dissemination behavior. Second, as Twitter is considered as a rich database for collecting individual health-related information to examine sentiments and manage public health [64] and reports highlight that individuals may be concerned about web-based interactions with peers [65], Twitter would be the best research context to meet the goals of this study. Third, this study distinguished between general and specific health information. Thus, we could offer more insights about privacy concern levels and disclosure behaviors related to the 2 types of health information on Twitter. These 3 reasons can make our study different from previous studies in the privacy literature.

In addition, we controlled for several variables such as age, gender, education, Twitter experience, privacy violation experience, and misrepresentation of identity on Twitter. According to previous studies in the privacy concern domain, some demographics, such as age [66], gender [67], and education level [68], can affect people’s intention to disclose information on social media. Moreover, the impacts of these variables have been examined in previous studies investigating individuals’ perceptions about sharing eHealth-related information [69,70]. The effects of these variables are often controlled in previous studies in the field of information privacy threats [71]. Thus, we assumed that individuals of different ages, genders, and educational levels engage in various disclosure behaviors because they have diverse backgrounds, individual characteristics, and personal differences. Therefore, we considered these demographics to be control variables in the proposed research model.

Moreover, the effects of misrepresentation of identity, experience with technology, and privacy violation experiences are controlled in previous studies examining relationships between privacy concerns and self-disclosure [22,42]. Thus, it is believed that individuals with different privacy violation experiences, previous identity misrepresentation, and experiences with Twitter are more likely to demonstrate various disclosure behaviors. Therefore, we treated these experience-related variables as control variables in our model.

## Methods

### Research Approach and Survey Development

We administered a web-based survey questionnaire to achieve the defined objectives and test the proposed model and research hypotheses. The survey consists of 4 sections. In the first part, the purpose of the study is described clearly, and a qualifying question is used to select respondents. The question for filtering respondents attempts to screen individuals with a Twitter account. Thus, individuals without a Twitter account are excluded from data collection and analysis. In the second section, respondents are asked to express their perceptions about privacy concerns associated with companies and third parties, peer-related privacy concerns, and health information dissemination behaviors. In the third section, demographic questions (ie, age, gender, education, income, and race) are asked. Finally, the last section focuses on personal privacy experiences (ie, Twitter experience, privacy violation experience, and misrepresentation of identity).

Questions to measure each construct were adapted from validated instruments available in the existing literature. Slight changes in the wording were made to fit the context of this study. We adapted items to measure CFIP (as a second-order construct with 4 dimensions) from the study by Stewart and Segars [40]. Following Zhang et al [22], we also conceptualized and measured PrPC as a second-order reflective construct with 4 dimensions. Previously defined scales to measure general and specific health information disclosure were adapted from the study by Hsu et al [72]. Respondents rated all the measuring items included in the survey using a 5-point Likert scale ranging from 1 (strongly disagree) to 5 (strongly agree). Multimedia Appendix 1 shows the questions used in the web-based survey.

### Data Collection and Data Analysis

Data were collected in April 2022 by uploading the questionnaire to Amazon’s Mechanical Turk (MTurk). MTurk is a crowdsourcing platform that enables researchers to access data from potential target samples to conduct a study. MTurk has been recognized as an acceptable web-based means for collecting individual-level data. Literature about health care analytics shows a growing number of studies using MTurk for health-related research [73]. Previous studies highlight that MTurk can measure individual perceptions in various domains, such as social media [74]. As the target population of this study was US citizens who use Twitter for web-based interactions, we limited the respondents’ location to the United States. Moreover, 2 attention-check questions were used to remove participants who chose answers without correctly replying to reverse-coded filler items [75]. The filtering questions were as follows: (1) It does not bother me that my peers may try to influence me through comments on my health-related postings on Twitter and (2) I am not concerned that I have little control over who can start a health-related conversation with me on Twitter. We received 364 questionnaires and excluded 35 (9.6%) that were either incomplete or failed the response quality questions, resulting in 329 (90.4%) valid and usable responses. The average response time to complete the questionnaire was 12 minutes. The descriptive statistics for demographics were performed using SPSS (version 26; IBM). The research model was tested using AMOS (version 26; IBM) within the structural equation model framework.

### Ethical Considerations

The institutional review board of Florida International University reviewed and approved the study (approval 112755). According to the institutional review board approval, written informed consent to participate in the study was obtained from all participants. Moreover, the data collected in this study were anonymous. We considered US $1 as an incentive for each respondent to participate in the study.

## Results

### Instrument Validation

We used confirmatory factor analysis to assess convergent and discriminant validity. Table 1 shows the results of the convergent validity test. The standardized factor loadings for all constructs exceeded 0.7, which is the acceptable range for factor loadings [76]. The composite reliability values and Cronbach α values were above the recommended value of .7, demonstrating the adequate reliability of the constructs [77]. All the values of average variance extracted (AVE) exceeded 0.5, which is the cutoff value [78]. These measures indicated the acceptability of the measurement model’s convergent validity.

Table 2 shows the discriminant validity of the constructs. All diagonal values (square roots of the AVEs) were >0.7 and greater than off-diagonal values (correlations) between any pair of constructs [79]. Thus, the discriminant validity requirements were satisfied for the research model.

Moreover, we checked the convergent and discriminant validity of the second-order constructs. The composite reliability, Cronbach α, and AVE values for CFIP were 0.91, .88, and 0.64, respectively, and these measures for PrPC were 0.94, .89, and 0.72, respectively. The correlation between the second-order variables (eg, CFIP and PrPC) was 0.58. Finally, the square roots of the AVEs for both constructs were >0.7 and higher than the correlations between the constructs. These results confirm an acceptable convergent and discriminant validity for both second-order constructs in the model.

**Table 1:** Results of the convergent validity test.

Constructs, subdimensions, and items				Standardized factor loading (>0.7)		Composite reliability (>0.7)		Cronbach α (>.7)		AVE^a^ (>0.5)
**Concern for information privacy**										
	**COLL^b^**					0.89		.75		0.66
		COLL1	0.82							
		COLL2	0.80							
		COLL3	0.81							
		COLL4	0.84							
	**USU^c^**					0.90		.79		0.70
		USU1	0.86							
		USU2	0.84							
		USU3	0.80							
		USU4	0.85							
	**IAC^d^**					0.86		.79		0.67
		IAC1	0.80							
		IAC2	0.84							
		IAC3	0.83							
	**ERR^e^**					0.89		.84		0.66
		ERR1	0.82							
		ERR2	0.84							
		ERR3	0.80							
		ERR4	0.81							
**Peer privacy concern**										
	**PPC^f^**					0.88		.84		0.66
		PPC1	0.82							
		PPC2	0.82							
		PPC3	0.80							
		PPC4	0.81							
	**CPC^g^**					0.89		.88		0.67
		CPC1	0.85							
		CPC2	0.82							
		CPC3	0.81							
		CPC4	0.80							
	**VTPC^h^**					0.89		.87		0.68
		VTPC1	0.85							
		VTPC2	0.80							
		VTPC3	0.84							
		VTPC4	0.82							
	**Peer-related information privacy concern–SSIPC^i^**					0.87		.82		0.63
		SSIPC1	0.79							
		SSIPC2	0.78							
		SSIPC3	0.81							
		SSIPC4	0.80							
	**Peer-related information privacy concern–PSIPC^j^**					0.89		.87		0.67
		PSIPC5	0.83							
		PSIPC6	0.79							
		PSIPC7	0.80							
		PSIPC8	0.86							
**GHID^k^**										
	**N/A^l^**					0.87		.84		0.64
		GHID1	0.81							
		GHID2	0.78							
		GHID3	0.82							
		GHID4	0.79							
**SHID^m^**										
	**N/A**					0.90		.88		0.69
		SHID1	0.84							
		SHID2	0.82							
		SHID3	0.82							
		SHID4	0.85							

^a^AVE: average variance extracted.

^b^COLL: collection.

^c^USU: unauthorized secondary use.

^d^IAC: improper access.

^e^ERR: error.

^f^PPC: psychological privacy concern.

^g^CPC: communication privacy concern.

^h^VTPC: virtual territory privacy concern.

^i^SSIPC: self-shared information privacy concern.

^j^PSIPC: peer-shared information privacy concern.

^k^GHID: general health information disclosure.

^l^N/A: not applicable.

^m^SHID: specific health information disclosure.

**Table 2:** Results of the discriminant validity test.

Construct	Score, mean (SD)	CFIP-COLL^a^	CFIP-USU^b^	CFIP-IAC^c^	CFIP-ERR^d^	PrPC-PPC^e^	PrPC-CPC^f^	PrPC-VTPC^g^	PrPC-PRIPC^h^	GHID^i^	SHID^j^
CFIP-COLL	4.03 (0.66)	*0.81* ^k^	—^l^	—	—	—	—	—	—	—	—
CFIP-USU	4.01 (0.67)	0.71	*0.83*	—	—	—	—	—	—	—	—
CFIP-IAC	3.94 (0.77)	0.62	0.66	*0.81*	—	—	—	—	—	—	—
CFIP-ERR	3.85 (0.82)	0.67	0.69	0.75	*0.81*	—	—	—	—	—	—
PrPC-PPC	3.92 (0.78)	0.32	0.35	0.17	0.43	*0.81*	—	—	—	—	—
PrPC-CPC	3.86 (0.88)	0.24	0.39	0.33	0.36	0.68	*0.81*	—	—	—	—
PrPC-VTPC	3.83 (0.86)	0.30	0.28	0.32	0.31	0.74	0.72	*0.82*	—	—	—
PrPC-PRIPC	3.87 (0.81)	0.19	0.36	0.31	0.33	0.69	0.70	0.74	*0.80*	—	—
GHID	3.84 (0.85)	0.34	0.32	0.42	0.36	0.35	0.42	0.30	0.49	*0.80*	—
SHID	3.81 (0.91)	0.30	0.30	0.38	0.31	0.37	0.38	0.41	0.41	0.53	*0.83*

^a^CFIP-COLL: concern for information privacy–collection.

^b^CFIP-USU: concern for information privacy–unauthorized secondary use.

^c^CFIP-IAC: concern for information privacy–improper access.

^d^CFIP-ERR: concern for information privacy–error.

^e^PrPC-PPC: peer privacy concern–psychological privacy concern.

^f^PrPC-CPC: peer privacy concern–communication privacy concern.

^g^PrPC-VTPC: peer privacy concern–virtual territory privacy concern.

^h^PrPC-PRIPC: peer privacy concern–peer-related information privacy concern.

^i^GHID: general health information disclosure.

^j^SHID: specific health information disclosure.

^k^Italicization represents the square roots of the average variance extracted.

^l^Not applicable.

### Respondents’ Characteristics

Table 3 shows the participants’ characteristics. The descriptive statistics demonstrate that respondents were fairly distributed across gender, where 52.9% (174/329) were men and 47.1% (155/329) were women. The age range was positively skewed, indicating that most participants were young, with a range between 25 and 34 years (155/329, 47.1%) being high, followed by the range between 35 and 44 years (102/329, 31%). Approximately half (178/329, 54.1%) of the respondents had undergraduate or graduate education levels, which aligns with previous studies highlighting that people with high education levels tend to search more often for web-based health information [80]. The annual income was fairly distributed, with income between US $60,000 and US $79,999 showing a high range (135/329, 41%) among the provided categories. Most respondents were White (174/329, 52.9%), followed by Hispanic and African American individuals. Approximately half (174/329, 52.9%) of the respondents reported using Twitter for 4 to 6 years. Overall, 52% (171/329) of the respondents indicated that they had a privacy violation experience at least once (for instance, their account was hacked), and 38.9% (128/329) mentioned that they tried to use a fake account on Twitter (at least once).

**Table 3:** Descriptive statistics (N=329).

Variables			Participants, n (%)
**Gender**			
	Men	174 (52.9)	
	Women	155 (47.1)	
**Age group (y)**			
	18-24	13 (4)	
	25-34	155 (47.1)	
	35-44	102 (31)	
	45-54	33 (10)	
	55-64	20 (6.1)	
	≥65	7 (2.1)	
**Education level**			
	Elementary	10 (3)	
	High school	66 (20.1)	
	College	76 (23.1)	
	Undergraduate	105 (31.9)	
	Graduate	72 (21.9)	
**Annual income (US $)**			
	<20,000	23 (7)	
	20,000-39,999	59 (17.9)	
	40,000-59,999	49 (14.9)	
	60,000-79,999	135 (41)	
	80,000-99,999	46 (14)	
	≥100,000	16 (4.9)	
**Race**			
	African American	36 (10.9)	
	Asian	23 (7)	
	Hispanic	66 (20.1)	
	Native American	13 (4)	
	White	174 (52.9)	
	Mixed	16 (4.9)	
**Twitter experience (y)**			
	1-3	63 (19.1)	
	4-6	174 (52.9)	
	7-9	66 (20.1)	
	10-12	20 (6.1)	
	>12	7 (2.1)	
**Privacy violation experience (eg, being hacked)**			
	Yes	171 (52)	
	No	158 (48)	
**Identity misrepresentation (eg, using fake accounts)**			
	Yes	201 (61.1)	
	No	128 (38.9)	

### Analysis of the Dimensions

When implementing second-order variables in a measurement model, there are 2 common approaches: the repeated items approach and the 2-step approach [81]. This study used a repeated items approach to measure reflective second-order constructs. In the repeated items approach, the indicators used to measure the second-order construct are included in the measurement model twice: once as indicators of the second-order construct and once as indicators of the corresponding first-order constructs [82]. This approach allows for a direct assessment of both the second-order and underlying first-order constructs in a single measurement model. The repeated items approach provides a holistic view of the measurement model by simultaneously assessing the second-order construct and its underlying dimensions [83]. Using the repeated items approach provides a more integrated perspective about how CFIP and PrPC are influenced by their respective first-order constructs. It allows for a direct examination of the relationships between the second-order construct and its underlying factors.

Both CFIP and PrPC are conceptualized as second-order reflective constructs, consistent with existing literature. A reflectively measured construct shares a common theme across subdimensions; the dimensions are expected to be highly correlated [84]. Table 2 shows that, consistent with reflective measurement, the 4 dimensions of CFIP (ie, collection, unauthorized secondary use, improper access, and errors) are highly correlated with each other. As expected, the 4 dimensions of PrPC (ie, psychological, communication, virtual territory, and peer-related information privacy concerns) are also highly correlated. Results show that the 4 dimensions of CFIP, as first-order factors, load significantly on the second-order construct. The loadings were 0.91 for collection, 0.80 for unauthorized secondary use, 0.95 for improper access, and 0.88 for errors. Therefore, the interaction among 4 dimensions reflects CFIP, which shares a common theme of losing control over information privacy owing to companies’ sharing behaviors. Furthermore, the 4 dimensions of PrPC also load significantly on the second-order construct. The loadings were 0.90 for psychological privacy concerns, 0.92 for communication privacy concerns, 0.86 for virtual territory privacy concerns, and 0.95 for peer-related information privacy concerns. Thus, interactions among these 4 dimensions represent PrPC, which exhibits a shared theme of privacy concerns about losing personal control owing to web-based peer behaviors.

### Structural Model and Path Analysis

Consistent with privacy literature, we controlled variables such as age, gender, education, years of experience, privacy violation experience, and misrepresentation of identity in the structural model [42]. Findings demonstrate that when the control variables are present, the coefficients and *R*^2^ change significantly. Specifically, when age (β=−.12; *P*=.01, education (β=.19; *P*=.003), and privacy violation experience (β=−.58; *P=*.008) are present in the model, they significantly influence health information disclosure. Thus, the findings confirm that young people with high education levels who have not experienced privacy violations are more likely to disclose health information on Twitter. However, no effects of gender, years of experience, and misrepresentation of identity were found on health information–sharing behaviors. We used the structural equation model technique** **to analyze the factors affecting health information disclosure on Twitter. The results of model fit indexes exhibit a good fit with the goodness-of-fit indexes (*χ*^2^_353_=2.2; goodness-of-fit index=0.84; adjusted goodness-of-fit index=0.81; comparative fit index=0.90; normed fit index=0.91; incremental fit index=0.90; standardized root mean square residual=0.03; and root mean square error of approximation=0.04) where all indexes meet their recommended cutoff values [85]. Table 4 depicts the summary of path analysis for 4 hypotheses (ie, H1A, H1B, H2A, and H2B).

**Figure 2:**
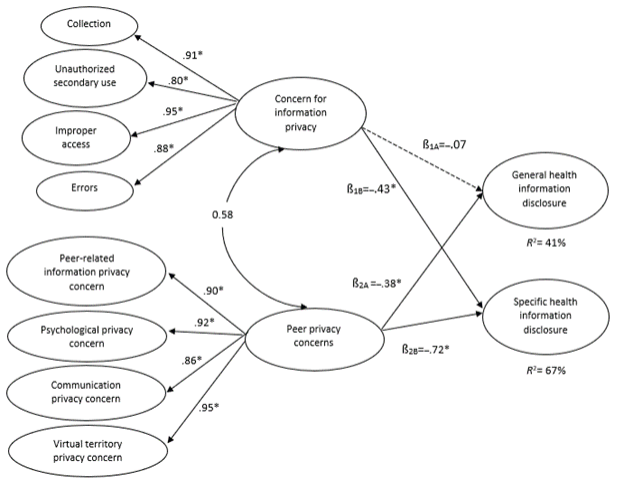
Model paths. H1A: hypothesis 1A; H1B: hypothesis 1B; H2A: hypothesis 2A; H2B: hypothesis 2B; *P<.001.

Figure 2 shows the standardized path coefficients of the structural model. Support is not found for H1A, which proposes that CFIP significantly influences general health information disclosure on Twitter (β=−.07; *P*=0.16). In contrast, the findings support H1B by confirming that CFIP significantly attenuates sharing behaviors when disclosing specific health information on Twitter (β=−.43; *P*<.001). H2A, which posits that PrPC would directly affect the disclosure of general health information on Twitter, is supported (β=−.38; *P*<.001). The analysis also exhibits that PrPC negatively shapes the sharing of specific health information on Twitter (β=−.72; *P*<.001), and this significant relationship supports H2B.

Regarding H1C and H2C, an alternative model was created for each hypothesis, and the 2 relationships in that hypothesis were constrained [86]. Next, a 2-tailed *t* test was used to compare the difference between the alternative and the original model. H1C posits a significant difference between the impact of CFIP on general and specific information–sharing behaviors. As the *t* value was significant (*t*_165_=3.45; *P*<.001), we confirm that CFIP imposes a more negative effect on specific health information dissemination than on sharing general health information on Twitter. In addition, H2C proposes that people may disclose more general health information than specific health information owing to peer-related privacy concerns. The *t* value was significant (*t*_165_=4.72; *P*<.001); thus, the effect of PrPC was more prominent in specific health information sharing than in disclosing general health information on Twitter.

Finally, the model explains 41% of the variance in general health information disclosure and 67% in specific health information sharing on Twitter. The *R*^2^ scores suggest that the 2 aspects of information privacy concerns (ie, concerns about the web-based practices of companies and peers’ behaviors) can provide reliable explanatory power to predict the variance in sharing general and specific health information.

**Table 4:** Path analysis.

Hypothesis	Path	Standardized coefficient, β	SE	Critical ratio	Results
1A	CFIP^a^–general health information disclosure	−.07	0.04	1.54	Not supported
1B	CFIP–specific health information disclosure	−.43^b^	0.03	4.21	Supported
2A	PrPC^c^–general health information disclosure	−.38^b^	0.05	5.37	Supported
2B	PrPC–specific health information disclosure	−.72^b^	0.05	7.12	Supported

^a^CFIP: concern for information privacy.

^b^Significance level, *P*<.001.

^c^PrPC: peer privacy concern.

## Discussion

### Principal Findings

Information sharing is one of the most important objectives of social media. People use Twitter for conversation, and information sharing can initiate a web-based exchange of ideas about an issue. As health information is more sensitive than other types of personal information, disclosing such data can raise privacy concerns. Most previous studies have mainly focused on privacy concerns related to companies and vendors as they may collect and use individuals’ personal information for other purposes or may not properly protect the collected information [87]. Few studies have also explained privacy concerns related to the web-based behaviors of peers [22]. However, previous literature did not consider both sides of information privacy concerns in a model in the context of social media. Moreover, disclosure behaviors on social media can be contingent upon the type of health information owing to sensitivity levels. Few studies have examined the sharing behaviors based on the unique characteristics of general and specific health information [86].

Although both antecedents (CFIP and PrPC) have been examined separately in previous studies, this study’s findings could propose scientific novelty. The study differs from previous research in this field because we integrated 2 aspects of privacy concerns (eg, related to companies and peers) to investigate the disclosure of general and specific health information on Twitter. In this study, we examined whether both aspects of information privacy concerns can jointly influence sharing decisions related to health-related information or whether the effect of one aspect can be overshadowed by the other; for instance, whether the nature of the relationships and contexts with 2 different information trustees (ie, vendors and peers) can influence people to share their health information on Twitter. The findings indicate that privacy concerns related to companies play a more significant role in predicting specific health information than in predicting general health information. Privacy concerns related to companies’ practices reflect the collection and misuse of health information by vendors, such as concerns about using health information for data mining and research purposes [88]. Our findings demonstrate that Twitter users are more concerned about vendors and companies using or sharing their personal health information than general health information. Thus, the dimensions of CFIP (ie, collection, unauthorized secondary use, improper access, and errors) become salient only when people want to disclose specific health information (such as information about their chronic diseases, signs, and symptoms or personal health status). Sharing public health information about hospitals, medical costs, and medications is not significantly affected by concerns about how companies may use such information. A plausible justification is that general health information cannot reflect any personal information associated with an individual, and even if it is used for data mining or big data analysis, it will not violate the user’s privacy needs. Consistent with previous studies [89], individuals may deliberately want to share general health information on social media to increase public awareness and knowledge about a medical situation, such as COVID-19 symptoms and vaccination. Regardless of information accuracy or misinformation, users may engage in sharing their general medical knowledge and public information about treatment options with almost no or minor privacy concerns related to companies and vendors’ collection and use practices.

Our results also show that peer-related privacy concerns can significantly shape both general and specific health information sharing on Twitter. Although Twitter is not the same as web-based health communities designed to share health information, many individuals use tweets to share personal and public health information [90]. Web-based interactions with peers may affect their sharing behaviors as they may feel unable to control who can see, comment on, or exchange the health information they share on Twitter. The dimensions of PrPC (ie, psychological, communication, virtual territory, and peer-related information privacy concerns) are important factors in predicting how users may disclose public and personal health information. However, peer-related privacy concerns are more intense for sharing personal than general health information. This finding indicates that when a user wants to share public information about a disease (for instance, cancer, COVID-19, or HIV), they are still concerned about how peers relate such general information to their profile. This concern becomes more salient when the user decides to reveal personal health information about that disease, such as what treatments or medications they are using daily or what medical procedures they will undergo in the future. Previous studies associate sharing personal health information related to physical health problems or mental disorders on web-based P2P networks with stigma [91]. The more sensitive the health information, the more stigma is attached to sharing such information with peers. Thus, being judged by peers (close friends and other web-based users) because of sharing personal health information may prevent them from disclosing that content on Twitter.

Although results show significant impacts of both aspects of privacy concerns on sharing specific health information on Twitter, peer-related privacy concerns are leading factors in shaping personal health information disclosure, more so than privacy concerns associated with companies and third parties. This result confirms the importance of web-based interactions with peers on social media and how to deal with their sharing behaviors, such as commenting or tagging [60]. This finding implies the critical effects of Twitter friends and the circle of people who can see and share tweets about private health information. Individuals may be first concerned about the Twitter circle and how peers would react to the shared personal information about health status and then become worried about how many companies may access such data and how they would use or share them. Thus, secondary dissemination of personal health information by web-based peers through liking, reposting, retweeting, or commenting on posts is more challenging to the maintenance of privacy controls than secondary use of data or unauthorized access to such private information by companies and vendors. Our finding that peer-related privacy concerns have a strong impact on health information sharing compared with privacy concerns associated with companies and third parties offers a counternarrative to prevalent assumptions in digital privacy research. This could be attributed to Twitter’s highly interactive and public nature, which might accentuate peer-related concerns. Previous studies, mainly those conducted in the context of web-based shopping or general social media use, might have overestimated the role of concerns associated with companies and third parties owing to the commercial and private nature of these web-based activities.

### Theoretical Implications

This study may offer some theoretical contributions. First, our findings have implications for information privacy research in social media by integrating the existing privacy concern perspectives. This study can open up the discussions through which privacy needs related to companies, third parties, and peer-related aspects can be addressed. Then, this comprehensive mechanism may strongly affect users’ sharing behavior patterns. Second, this study distinguishes the differences between sharing public and private health information on Twitter. Although disclosing specific health information may help share personal experiences related to various medical situations that could be useful for peers, it is more challenging than disseminating general health information. The findings demonstrate how company-related and peer-related privacy concerns could shape the 2 types of information-sharing behaviors. Third, this study investigates the effects of information privacy mechanisms on health information sharing in the context of Twitter. The findings can promote discussions about health information disclosure in other P2P networks such as other social media platforms, virtual worlds, or Metaverse. Fourth, exploring the determinants of information sharing regarding different types of privacy concerns can expand our current understanding of knowledge acquisition. As sharing both general and specific health information on social media can contribute to people’s medical knowledge, addressing the barriers to specific health information sharing and removing the privacy challenges can significantly help the procedures of medical knowledge acquisition from web-based interactions with peers.

The study contributes significantly to the theoretical understanding of privacy concerns in web-based health information sharing. The evidence that peer-related privacy concerns influence more strongly than those related to companies and third parties highlights a potential oversight in theoretical perspectives. Current theories largely view companies as the predominant source of digital privacy concerns, and this may need re-evaluation. The results extend existing theories by emphasizing the role of peer interactions in privacy concerns, particularly in public and highly interactive web-based environments such as Twitter. This recognition of the social dimension of privacy concerns could be integrated into existing theoretical models to provide a more comprehensive framework for web-based privacy behavior. Furthermore, although our study is specific to Twitter and health information, the insights gained may have broad applicability. The potential role of peer-related privacy concerns could be a valuable area of exploration in other social media contexts and in sharing other types of sensitive information. Thus, our findings open up new avenues for theoretical exploration and suggest a need for further studies to fully understand the complexities of privacy behavior in the digital age.

Unlike other research approaches, such as experiments, observational data, or qualitative interviews to assess privacy concerns and information sharing, our study used a quantitative survey approach. This allowed us to capture data from a large and more diverse sample, providing a more robust and generalizable understanding of privacy concerns in web-based health information sharing. The strength of our quantitative approach lies in its ability to establish clear patterns and relationships among various factors influencing privacy concerns. This enabled us to derive a more comprehensive and systematic understanding of the factors that significantly influence privacy concerns and health information sharing on Twitter. In terms of comparison, our findings offer a novel perspective about the role of peer-related privacy concerns in shaping web-based health information–sharing behaviors. Previous studies have predominantly focused on company-related and third party–related privacy concerns. However, our study highlighted the paramount influence of peer-related privacy concerns, thus suggesting a reorientation of focus in subsequent research efforts in this area. Our study also provides quantifiable evidence about the relative influence of peer-related privacy concerns and such privacy concerns associated with companies and third parties on Twitter users’ health information–sharing behaviors. Such quantifiable insights could serve as valuable benchmarks for future studies seeking to measure and compare similar variables in different contexts or on different platforms. Our survey methodology, coupled with a comparative analysis of the findings, underscores the contribution of our study to the field, offering both nuanced insights and broad trends that enrich our understanding of privacy concerns and health information sharing on social media platforms.

### Practical Contributions

This study also provides several practical and technical implications for promoting privacy protection on Twitter. To promote the sharing of specific health information, it is essential to address privacy concerns related to both companies and peers. However, addressing peer-related privacy concerns is vital to encourage the disclosure of general health information. This is because concerns related to companies and third parties do not significantly predict general health information sharing. Thus, a robust privacy policy cannot be developed regardless of information type. As the 2 types of health information require different ways of satisfying privacy needs, mechanisms and regulations facilitating general and specific health information sharing cannot be the same. Depending on the type of health information, it is essential to customize the ability of Twitter users to control their self-concept and meet different privacy protection requirements. General procedures and privacy policies to regulate the dissemination and use of personal posts are not sufficient to address the information privacy concerns. Twitter should allay users’ privacy concerns about sharing specific health information using advanced technology and management mechanisms. For example, Twitter can enable individuals to restrict access to their shared personal health information. Punitive regulations can be established for inappropriate behaviors (such as retweeting without consent) that may discourage sharing specific health information. All controlling mechanisms and privacy protection functionalities should be easy to understand and use and should not be an additional burden on the users.

As the 2 aspects (company-related and peer-related aspects) of privacy concerns manifest in several dimensions, different features can be developed to address the need for effective protection mechanisms. Twitter can add a new feature to tweets, enabling users to identify the sensitivity of posts related to health information. The content will be recognized as a private post if the sensitivity score (eg, calculated based on a scale ranging from 1 to 10) is more than average. Then, that post is automatically restricted from exposure to everyone, and users can share their thoughts and experiences only with a small crowd. Users can also define terms and conditions for peers who want to retweet sensitive posts. For instance, a “request for share” button can appear for each sensitive post, and peers can only share the posts when they get approval from the focal users. Given our findings, Twitter could introduce a feature that allows users to select the audience for their health-related posts, thereby addressing peer-related privacy concerns. They could also introduce a “Health Information” mode that automatically applies high privacy settings for tweets marked as health related. In addition, given the significant role of knowledge in shaping privacy concerns, Twitter should consider educational campaigns or prompts to inform users about these features and the importance of privacy when sharing health information.

In May 2022, Elon Musk called for further investigation into the accuracy of spam and fake account estimates, which Twitter announced to be <5%. Fake and spam accounts could lead to undesirable social interactions with peers, unwanted peer-shared information, and an unpleasant web-based social environment. Twitter needs to use new procedures to detect spam and fake accounts and better control the functionality of Twitter bots to provide a more appropriate web-based social platform. This new mechanism could automatically limit the visibility of private posts containing highly sensitive health information to everyone, even to people who users follow. On the basis of the current Twitter privacy policy, people can mention who can reply to a specific tweet. However, it is hard to confirm who can actually see the posts because of bots and recommendation agents.

Social bots use computer algorithms to artificially create content and interact with people on social media [92]. Twitter bots can be manipulative and purposely change people’s attitudes and opinions about a topic [93]. For instance, bots can share posts with peers who are not following a user but usually read posts with health information content. The existence of bots may be useful for sharing general information but can be very harmful because it can increase exposure to private posts with sensitive health information. A plausible recommendation is to add a new category for sensitive content (such as specific health information) besides the photo, graphics interchange format (GIF), and poll categories. Then, users can create a new circle of people who are allowed to see, reply to, or share these sensitive posts. Users can also customize the configuration and limit the possible unwanted interactions by selecting who can see the posts but cannot share them. This small crowd can be saved for future use and can be easily modified later. The advantage of this new category is that people are notified to customize their Twitter circle depending on different content (eg, highly sensitive, semisensitive, and nonsensitive). For instance, a user can select everyone to see and comment on posts containing information about cryptocurrency, high-technology companies, or humanitarian issues. In contrast, the user can select a group of followers to see their thoughts about general health information and choose only a few close friends to see and comment on posts with sensitive health information.

Stringent privacy policies are required to enable Twitter users to limit who (ie, peers) can view, comment on, and share web-based content. People should be able to easily edit with whom they can share health information to exercise control over their personal digital information. Spambots on Twitter should also be controlled, modified, or filtered because they can involve potentially deceptive, harmful, or annoying activities. A more transparent policy is required to detect and deactivate invasive Twitter bots that can automatically like or retweet users’ postings without consent.

Finally, the insights from our study are not only limited to Twitter but also have implications for other social media platforms where users might share health information. Such platforms should recognize the significant role of peer-related privacy concerns and consider introducing similar audience control features. Health professionals and health-related organizations using social media for patient engagement should also be aware of these concerns and take steps to ensure that their communication respects patient privacy. Policy makers should consider our findings when developing regulations for health information sharing on social media to ensure that they address the most significant privacy concerns.

### Limitations and Future Studies

Our study also has some limitations that can be considered as opportunities for future studies. First, a web-based survey through MTurk was used to collect data, which may be biased toward people familiar with crowdsourcing platforms. Future studies can use other data collection and sampling strategies, such as collecting data directly from Twitter. Second, we collected data from 329 Twitter users, which may not be a good representative of Twitter users. Next, studies can increase the sample size to reduce sampling bias and improve the generalizability of the findings. Third, we did not consider the effects of cultural dimensions (such as individualism, uncertainty avoidance, etc) on sharing health information on Twitter. It can be interesting for future studies to explore the effect of culture on disclosing different types of health information on social media. Fourth, our study tests a model to analyze health information sharing from the perspective of privacy concerns. However, there may be other essential variables. More studies are required to examine other factors inhibiting and promoting sharing behaviors on social media, such as reputation, incentives, trust, stigma, and social support. Fifth, this study did not examine the accuracy of the health information shared on Twitter or the risks of misinformation because it is not within the scope of this study. Future studies could expand upon our results to investigate the role of misinformation risks in information-sharing behaviors. Finally, our study focused on Twitter as a study context. We encourage future studies to extend the proposed model to other social media platforms (eg, Facebook, TikTok, and Instagram) where web-based interactions with peers are essential.

### Conclusions

This study provides insights into health information sharing on Twitter from a privacy perspective. The findings propose that including CFIP and PrPC constructs can help in better conceptualization of information privacy concerns in the context of social media. The integration of these 2 aspects of information privacy can expand the discussion about internet privacy by addressing the privacy needs associated with the practices of companies, such as collection, unauthorized secondary use, improper access, and errors. It also considers psychological privacy concerns, communication privacy concerns, peers’ sharing behaviors, and territory privacy concerns related to peers in such interpersonal interactions. This interactive approach can provide a more comprehensive analysis of information privacy (related to web-based vendors and web-based peers) and adds a more substantial explanation of privacy needs on social media channels (such as Twitter). Privacy concerns may not always prohibit disclosure behaviors on Twitter; it depends on the type of health information. The findings demonstrate that peer-related privacy concerns are more salient to predicting general and specific health information sharing on Twitter than privacy concerns related to companies and third parties. The results could propose practical contributions by shedding more light on the negative impacts of web-based peer behaviors on losing personal control over digital communications and information access. Privacy policies should focus on companies’ practices, such as sharing users’ information with third parties for big data analytics. We suggest mitigating privacy concerns and promoting health information sharing on Twitter by creating policies that tailor privacy needs to the type of health information shared (ie, general or specific).

## Appendix

Multimedia Appendix 1 Web-based survey—measuring items.

## Abbreviations

AVE: average variance extracted
CDC: Centers for Disease Control and Prevention
CFIP: concern for information privacy
GIF: graphics interchange format
H1A: hypothesis 1A
H1B: hypothesis 1B
H1C: hypothesis 1C
H2A: hypothesis 2A
H2B: hypothesis 2B
H2C: hypothesis 2C
MTurk: Mechanical Turk
P2P: peer-to-peer
PrPC: peer privacy concern

## Data Availability

The data sets generated and analyzed during this study are available from the corresponding author upon reasonable request.

## References

[1] Benetoli A, Chen TF, Aslani P (2017). Consumer health-related activities on social media: exploratory study. J Med Internet Res.

[2] Alvarez-Mon MA, Asunsolo Del Barco A, Lahera G, Quintero J, Ferre F, Pereira-Sanchez V, Ortuño F, Alvarez-Mon M (2018). Increasing interest of mass communication media and the general public in the distribution of tweets about mental disorders: observational study. J Med Internet Res.

[3] Laranjo L, Arguel A, Neves AL, Gallagher AM, Kaplan R, Mortimer N, Mendes GA, Lau AY (2015). The influence of social networking sites on health behavior change: a systematic review and meta-analysis. J Am Med Inform Assoc.

[4] Moorhead SA, Hazlett DE, Harrison L, Carroll JK, Irwin A, Hoving C (2013). A new dimension of health care: systematic review of the uses, benefits, and limitations of social media for health communication. J Med Internet Res.

[5] Smith A, Anderson M (2018). Social media use in 2018. Pew Research Center.

[6] Corsi N, Nguyen DD, Butaney M, Majdalany SE, Corsi MP, Malchow T, Piontkowski AJ, Trinh QD, Loeb S, Abdollah F (2023). Top 100 urology influencers on Twitter: is social media influence associated with academic impact?. Eur Urol Focus.

[7] Ahmed W, Taft TH, Charabaty A (2021). Social media in inflammatory bowel disease: the patient and physician perspective. Curr Opin Gastroenterol.

[8] Saura JR, Palacios-Marqués D, Ribeiro-Soriano D (2023). Privacy concerns in social media UGC communities: understanding user behavior sentiments in complex networks. Inf Syst E-Bus Manage.

[9] Appari A, Johnson ME (2010). Information security and privacy in healthcare: current state of research. Intl J Internet Enterp Manage.

[10] Eichstaedt JC, Smith RJ, Merchant RM, Ungar LH, Crutchley P, Preoţiuc-Pietro D, Asch DA, Schwartz HA (2018). Facebook language predicts depression in medical records. Proc Natl Acad Sci USA.

[11] Schwartz HA, Eichstaedt J, Kern ML, Park G, Sap M, Stillwell D, Kosinski M (2014). Towards assessing changes in degree of depression through Facebook.

[12] Jiang K, Gupta R, Gupta M, Calix RA, Bernard GR (2017). Identifying personal health experience tweets with deep neural networks.

[13] Fish R, Hatton C, Chauhan U (2017). “Tell me what they do to my body”: a survey to find out what information people with learning disabilities want with their medications. Brit J Learn Disabil.

[14] Liu D, Brown BB (2014). Self-disclosure on social networking sites, positive feedback, and social capital among Chinese college students. Comput Hum Behav.

[15] Kshetri N (2014). Big data׳s impact on privacy, security and consumer welfare. Telecomm Policy.

[16] Price WN 2nd, Cohen IG (2019). Privacy in the age of medical big data. Nat Med.

[17] Ozdemir ZD, Smith HJ, Benamati JH (2018). Antecedents and outcomes of information privacy concerns in a peer context: an exploratory study. Eur J Inf Syst.

[18] Yeung AW, Kletecka-Pulker M, Eibensteiner F, Plunger P, Völkl-Kernstock S, Willschke H, Atanasov AG (2021). Implications of Twitter in health-related research: a landscape analysis of the scientific literature. Front Public Health.

[19] Sinnenberg L, Buttenheim AM, Padrez K, Mancheno C, Ungar L, Merchant RM (2017). Twitter as a tool for health research: a systematic review. Am J Public Health.

[20] Singh M, Bansal D, Sofat S (2018). Who is who on Twitter–spammer, fake or compromised account? a tool to reveal true identity in real-time. Cybern Syst.

[21] Smith HJ, Dinev T, Xu H (2011). Information privacy research: an interdisciplinary review. MIS Q.

[22] Zhang N, Wang C, Karahanna E, Xu Y (2022). Peer privacy concerns: conceptualization and measurement. MIS Q.

[23] Li J (2015). A privacy preservation model for health-related social networking sites. J Med Internet Res.

[24] Valdez D, Ten Thij M, Bathina K, Rutter LA, Bollen J (2020). Social media insights into US mental health during the COVID-19 pandemic: longitudinal analysis of Twitter data. J Med Internet Res.

[25] Grover P, Kar AK, Davies G (2018). “Technology enabled health” – insights from Twitter analytics with a socio-technical perspective. Int J Inf Manage.

[26] Chew C, Eysenbach G (2010). Pandemics in the age of Twitter: content analysis of tweets during the 2009 H1N1 outbreak. PLoS One.

[27] Chunara R, Andrews JR, Brownstein JS (2012). Social and news media enable estimation of epidemiological patterns early in the 2010 Haitian cholera outbreak. Am J Trop Med Hyg.

[28] Sarker A, O'Connor K, Ginn R, Scotch M, Smith K, Malone D, Gonzalez G (2016). Social media mining for toxicovigilance: automatic monitoring of prescription medication abuse from Twitter. Drug Saf.

[29] Reavley NJ, Pilkington PD (2014). Use of Twitter to monitor attitudes toward depression and schizophrenia: an exploratory study. PeerJ.

[30] Lee JL, DeCamp M, Dredze M, Chisolm MS, Berger ZD (2014). What are health-related users tweeting? a qualitative content analysis of health-related users and their messages on Twitter. J Med Internet Res.

[31] Scanfeld D, Scanfeld V, Larson EL (2010). Dissemination of health information through social networks: Twitter and antibiotics. Am J Infect Control.

[32] Lee I (2018). Social media analytics for enterprises: typology, methods, and processes. Bus Horiz.

[33] Valaitis RK, Akhtar-Danesh N, Brooks F, Binks S, Semogas D (2011). Online communities of practice as a communication resource for community health nurses working with homeless persons. J Adv Nurs.

[34] Ghosh DD, Guha R (2013). What are we 'tweeting' about obesity? mapping tweets with topic modeling and geographic information system. Cartogr Geogr Inf Sci.

[35] Prier KW, Smith MS, Giraud-Carrier C, Hanson CL (2011). Identifying health-related topics on Twitter.

[36] Guo JW, Radloff CL, Wawrzynski SE, Cloyes KG (2020). Mining Twitter to explore the emergence of COVID-19 symptoms. Public Health Nurs.

[37] Saniei R, Rodríguez Doncel V (2022). PHDD: corpus of physical health data disclosure on Twitter during COVID-19 pandemic. SN Comput Sci.

[38] (2011). Social media guidelines and best practices. Centers for Disease Control and Prevention.

[39] Medford RJ, Saleh SN, Sumarsono A, Perl TM, Lehmann CU (2020). An "infodemic": leveraging high-volume Twitter data to understand early public sentiment for the coronavirus disease 2019 outbreak. Open Forum Infect Dis.

[40] Stewart KA, Segars AH (2002). An empirical examination of the concern for information privacy instrument. Inf Syst Res.

[41] Rohunen A, Markkula J (2018). On the road – listening to data subjects’ personal mobility data privacy concerns. Behav Inf Technol.

[42] Malhotra NK, Kim SS, Agarwal J (2004). Internet users' information privacy concerns (IUIPC): the construct, the scale, and a causal model. Inf Syst Res.

[43] Li H, Sarathy R, Xu H (2011). The role of affect and cognition on online consumers' decision to disclose personal information to unfamiliar online vendors. Decis Support Syst.

[44] Smith HJ, Milberg SJ, Burke SJ (1996). Information privacy: measuring individuals' concerns about organizational practices. MIS Q.

[45] Jeong Y, Kim Y (2017). Privacy concerns on social networking sites: interplay among posting types, content, and audiences. Comput Hum Behav.

[46] Thackeray R, Neiger BL, Hanson CL, McKenzie JF (2008). Enhancing promotional strategies within social marketing programs: use of web 2.0 social media. Health Promot Pract.

[47] Liu B (2015). Sentiment Analysis: Mining Opinions, Sentiments, and Emotions.

[48] Gupta A, Katarya R (2020). Social media based surveillance systems for healthcare using machine learning: a systematic review. J Biomed Inform.

[49] Debatin B, Lovejoy JP, Horn AK, Hughes BN (2009). Facebook and online privacy: attitudes, behaviors, and unintended consequences. J Comput Mediat Commun.

[50] Nabity-Grover T, Cheung CM, Thatcher JB (2020). Inside out and outside in: how the COVID-19 pandemic affects self-disclosure on social media. Int J Inf Manage.

[51] Choi BC, Jiang Z, Xiao B, Kim SS (2015). Embarrassing exposures in online social networks: an integrated perspective of privacy invasion and relationship bonding. Inf Syst Res.

[52] Boshmaf Y, Muslukhov I, Beznosov K, Ripeanu M (2013). Design and analysis of a social botnet. Comput Netw.

[53] Fire M, Goldschmidt R, Elovici Y (2014). Online social networks: threats and solutions. IEEE Commun Surv Tutor.

[54] Zhang Y, Luo C, Wang H, Chen Y, Chen Y (2022). ‘A right to be forgotten’: retrospective privacy concerns in social networking services. Behav Inf Technol.

[55] Lin S, Armstrong D (2019). Beyond information: the role of territory in privacy management behavior on social networking sites. J Assoc Inf Syst.

[56] Frampton BD, Child JT (2013). Friend or not to friend: coworker Facebook friend requests as an application of communication privacy management theory. Comput Hum Behav.

[57] Lin HC, Chen YJ, Chen CC, Ho WH (2018). Expectations of social networking site users who share and acquire health-related information. Comput Electr Eng.

[58] Wang C, Zhang X, Hann IH (2018). Socially nudged: a quasi-experimental study of friends’ social influence in online product ratings. Inf Syst Res.

[59] Stella M, Ferrara E, De Domenico M (2018). Bots increase exposure to negative and inflammatory content in online social systems. Proc Natl Acad Sci U S A.

[60] Karahanna E, Xin Xu S, Xu Y, Zhang N (2018). The needs–affordances–features perspective for the use of social media. MIS Q.

[61] Rønn KV, Søe SO (2020). Is social media intelligence private? privacy in public and the nature of social media intelligence. Intelligence on the Frontier Between State and Civil Society.

[62] Esmaeilzadeh P (2019). The effects of public concern for information privacy on the adoption of health information exchanges (HIEs) by healthcare entities. Health Commun.

[63] Wang C, Zhang N, Wang C (2021). Managing privacy in the digital economy. Fundam Res.

[64] Sewalk KC, Tuli G, Hswen Y, Brownstein JS, Hawkins JB (2018). Using Twitter to examine web-based patient experience sentiments in the United States: longitudinal study. J Med Internet Res.

[65] Humphreys L, Gill P, Krishnamurthy B (2010). How much is too much? Privacy issues on Twitter.

[66] Christofides E, Muise A, Desmarais S (2012). Risky disclosures on Facebook: the effect of having a bad experience on online behavior. J Adolesc Res.

[67] Valkenburg PM, Sumter SR, Peter J (2010). Gender differences in online and offline self‐disclosure in pre‐adolescence and adolescence. Brit J Dev Psychol.

[68] Zou ML, Li MX, Cho V (2020). Depression and disclosure behavior via social media: a study of university students in China. Heliyon.

[69] Dimitropoulos L, Patel V, Scheffler SA, Posnack S (2011). Public attitudes toward health information exchange: perceived benefits and concerns. Am J Manag Care.

[70] O'Donnell HC, Patel V, Kern LM, Barrón Y, Teixeira P, Dhopeshwarkar R, Kaushal R (2011). Healthcare consumers' attitudes towards physician and personal use of health information exchange. J Gen Intern Med.

[71] Zhang X, Liu S, Chen X, Wang L, Gao B, Zhu Q (2018). Health information privacy concerns, antecedents, and information disclosure intention in online health communities. Inf Manag.

[72] Hsu MH, Ju TL, Yen CH, Chang CM (2007). Knowledge sharing behavior in virtual communities: the relationship between trust, self-efficacy, and outcome expectations. Int J Hum Comput Stud.

[73] Esmaeilzadeh P (2021). How does IT identity affect individuals’ use behaviors associated with personal health devices (PHDs)? an empirical study. Inf Manag.

[74] Chandler J, Shapiro D (2016). Conducting clinical research using crowdsourced convenience samples. Annu Rev Clin Psychol.

[75] Thomas KA, Clifford S (2017). Validity and mechanical turk: an assessment of exclusion methods and interactive experiments. Comput Hum Behav.

[76] Hair J, Black W, Babin B, Anderson R, Tatham R (2006). Multivariate Data Analysis, 6th edition.

[77] Chin WW, Marcoulides G (1998). The partial least squares approach to structural equation modeling. Adv Hosp Leis.

[78] Segars AH (1997). Assessing the unidimensionality of measurement: a paradigm and illustration within the context of information systems research. Omega.

[79] Fornell C, Tellis GJ, Zinkhan GM (1982). Validity assessment: a structural equations approach using partial least squares.

[80] Jo HS, Hwang MS, Lee H (2010). Market segmentation of health information use on the internet in Korea. Int J Med Inform.

[81] van Riel AC, Henseler J, Kemény I, Sasovova Z (2017). Estimating hierarchical constructs using consistent partial least squares. Ind Manag Data Syst.

[82] Duarte P, Amaro S (2018). Methods for modelling reflective-formative second order constructs in PLS: an application to online travel shopping. J Hosp Tour Technol.

[83] Carillo K, Scornavacca E, Za S (2017). The role of media dependency in predicting continuance intention to use ubiquitous media systems. Inf Manag.

[84] MacKenzie SB, Podsakoff PM, Podsakoff NP (2011). Construct measurement and validation procedures in MIS and behavioral research: integrating new and existing techniques. MIS Q.

[85] Hooper D, Coughlan J, Mullen M (2008). Evaluating model fit: a synthesis of the structural equation modelling literature.

[86] Yan Z, Wang T, Chen Y, Zhang H (2016). Knowledge sharing in online health communities: a social exchange theory perspective. Inf Manag.

[87] Xu H, Dinev T, Smith J, Hart P (2011). Information privacy concerns: linking individual perceptions with institutional privacy assurances. J Assoc Inf Syst.

[88] Koh H, Tan G (2005). Data mining applications in healthcare. J Healthc Inf Manag.

[89] Song H, Omori K, Kim J, Tenzek KE, Morey Hawkins J, Lin WY, Kim YC, Jung JY (2016). Trusting social media as a source of health information: online surveys comparing the United States, Korea, and Hong Kong. J Med Internet Res.

[90] De Choudhury M, Morris MR, White RW (2014). Seeking and sharing health information online: comparing search engines and social media.

[91] Naslund JA, Aschbrenner KA, Marsch LA, Bartels SJ (2016). The future of mental health care: peer-to-peer support and social media. Epidemiol Psychiatr Sci.

[92] Ferrara E, Varol O, Davis C, Menczer F, Flammini A (2016). The rise of social bots. Commun ACM.

[93] Domenico GD, Sit J, Ishizaka A, Nunan D (2021). Fake news, social media and marketing: a systematic review. J Bus Res.

