# Supplementary material for: Privacy Concerns About Sharing General and Specific Health Information on Twitter: Quantitative Study
Source: JMIR Form Res. 2024 Jan 12;8:e45573. doi: 10.2196/45573 (PMC10789368; doi:10.2196/45573)
Supplement: Multimedia Appendix 1 [file formative_v8i1e45573_app1.docx]

**Appendix:**

Online Survey- measuring items

| **Constructs** | **Measuring items** |
| --- | --- |
| CFIP: Collection | 1. It usually bothers me when companies ask me for health information.  2. When companies ask me for health information, I sometimes think twice about providing it.  3. It bothers me to give health information to so many companies.  4. I’m concerned that companies are collecting too much health information about me from my Twitter. |
| CFIP: Unauthorized secondary use | 1. I am concerned that companies would use health information from my Twitter for any purpose without my authorization  2. I am concerned that when I give health information to a company for some reason, the company will use the information for any other reason.  3. I am concerned that companies would sell my health information in their computer databases to other companies.  4. I am concerned that companies would share health information shared on Twitter with other companies even when I have not authorized it |
| CFIP: Improper access | 1. I am concerned that companies do not devote enough time and effort to preventing unauthorized access to health information shared on Twitter.  2. I am concerned that companies do not take enough steps to ensure that unauthorized people cannot access health information on their computers.  3. I am concerned that companies do not protect computer databases that contain health information shared on Twitter from unauthorized access. |
| CFIP: Errors | 1. I am concerned that companies do not double-check my health information stored in computer databases for accuracy  2. I am concerned that companies do not have adequate procedures to correct errors in my health information shared on Twitter.  3. I am concerned that companies do not devote more time and effort to verifying the accuracy of my health information in their databases.  4. I am concerned that companies do not take more steps to ensure that my health information in their files is accurate |
| PrPc: Psychological privacy concern | 1. It bothers me when my peers offer advice and opinions on Twitter about health-related areas that I want to determine for myself.  2. It bothers me that my peers may try to influence me through comments on my health-related postings on Twitter.  3. I am concerned that my health-related opinions are unduly influenced by postings from my Twitter peers.  4. I am concerned that how I feel about a health-related issue is influenced by postings and comments from my Twitter peers. |
| PrPc: Communication privacy concern | 1. It bothers me that I have little control over when my Twitter peers can start a health-related conversation with me on Twitter.  2. I am concerned that I have little control over how my Twitter peers can start an online health-related conversation with me on Twitter.  3. I am concerned that I have little control over which method (direct message, email, commenting on my post, etc.) my Twitter peers can use to contact me.  4. I am concerned that I have little control over who can start a health-related conversation with me on Twitter. |
| PrPc: Virtual territory privacy concern | 1. I am concerned that my Twitter peers may post unwanted health-related content on my Twitter Timeline.  2. I am concerned that my Twitter peers may post health-related contents that do not represent who I am on my Twitter Timeline.  3.I am concerned that my Twitter peers may post health-related contents that I do not want to be associated with me on my Twitter Timeline.  4. I am concerned that my Twitter friends may post embarrassing health-related content on my Twitter Timeline. |
| PrPc: Peer-related information privacy concern | Self-Shared Information Privacy Concern  1. It bothers me that I do not have control over how the health-related information I post on Twitter is used by my Twitter peers.  2. I am concerned that my Twitter peers can use the health-related information that I post on Twitter for other purposes.  3. I am concerned that my Twitter friends can unintentionally (through their “like,” “tagging,” and “sharing” activities) expose health-related pictures I post to people who are not in my “Friends” circle.  4. I am concerned that my Twitter friends can unintentionally (through their “like,” “tagging,” and “sharing” activities) expose my health-related postings to people who are not in my “Friends” circle.  Peer-Shared Information Privacy Concern  1. I am concerned that my Twitter friends can post embarrassing health-related information about me on Twitter.  2. I am worried that I may not have full control over who can post health-related information about me on Twitter.  3. I am concerned that my Twitter friends may post health-related information about me on Twitter that is not correct.  4. I am concerned that my Twitter friends may post inaccurate health-related information about me on Twitter. |
| General health information disclosure | 1: When participating on Twitter, I usually actively share some public information I know, including hospital information, medicine price, and so on.  2: When discussing problems related to hospitals, medicine, and other public health information, I am usually involved in the subsequent interactions on Twitter.  3: I usually spend a lot of time conducting general health information (e.g., hospital and medicine information) sharing activities on Twitter  4: I frequently participate in sharing general health information (e.g., hospital and medicine information) on Twitter |
| Specific health-information disclosure | 1: When participating in social media (e.g., Instagram), I usually actively share my personal health information, including treatment experience, health problems, and so on  2: When discussing problems related to medical treatment, medical experience, and other private health-related issues, I am usually involved in the subsequent interactions on Twitter.  3: I usually spend a lot of time conducting specific health information  (i.e., personal medical issues, my treatments) sharing activities on Twitter  4: I frequently participate in specific health information (i.e., personal  medical issues, my treatments) sharing activities on Twitter |
